# Supplementary material for: Biomarkers of pediatric Epstein-Barr virus-associated hemophagocytic lymphohistiocytosis through single-cell transcriptomics
Source: Nat Commun. 2025 Jul 25;16:6888. doi: 10.1038/s41467-025-62090-5 (PMC12297430; doi:10.1038/s41467-025-62090-5)
Supplement: Supplementary file 1 — Supplementary Information [file 41467_2025_62090_MOESM1_ESM.pdf]

Biomarkers of Pediatric Epstein-Barr Virus-Associated Hemophagocytic Lymphohistiocytosis through Single-Cell Transcriptomics

Jie Shen, Yunyan He, Hong Zheng, Jianwen Xiao, Fu Li, Keke Chen, Biyun Guo, Yulei He, Lin Liu, Zhi Lin, Dan Wang, Leping Liu, Shengfeng Wang, Wen Zhou, Yingchi Zhang, Jian Wei, Yunchu Wang, Rong Hu, Daolin Tang, Dao Wang, Minghua Yang

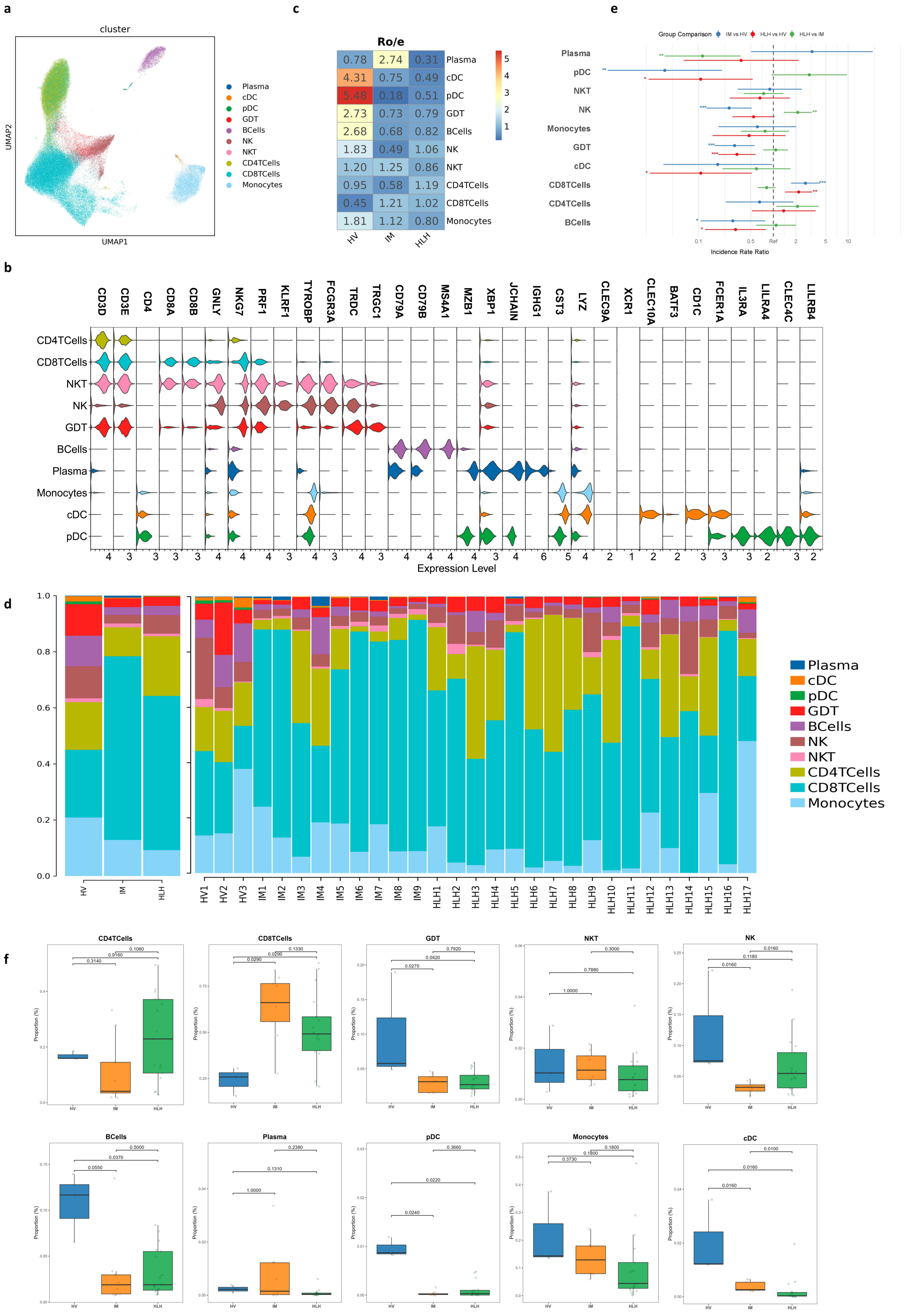

Supplementary Fig. 1: Overview of single-cell atlas. (a) UMAP visualization of 216,817 single cells from 29 samples. (b) Violin plot showing the expression patterns of canonical markers. Each column corresponded to specific marker genes, while rows denoted clusters, colored identically to those in a. (c) Quantification of cell subpopulation enrichment based on Ro/e for cellular populations. (d) Cell compositions at single group and single sample level. (e) The forest plot demonstrating intergroup comparisons of cell subtype counts. Specifically, a negative binomial regression model was employed, with the count of each cellular subset as the dependent variable and group classification as the independent variable. An offset term of log (total captured cell number per sample) was incorporated to control for potential confounding effects caused by variations in total cell capture efficiency across samples. *P* value was subjected to FDR corrected. (f) Box plot depicting the percentage of each cell cluster, with the median indicated by a horizontal line. Two-sided Wilcoxon tests were performed between groups and *P* values were adjusted using the BH correction.

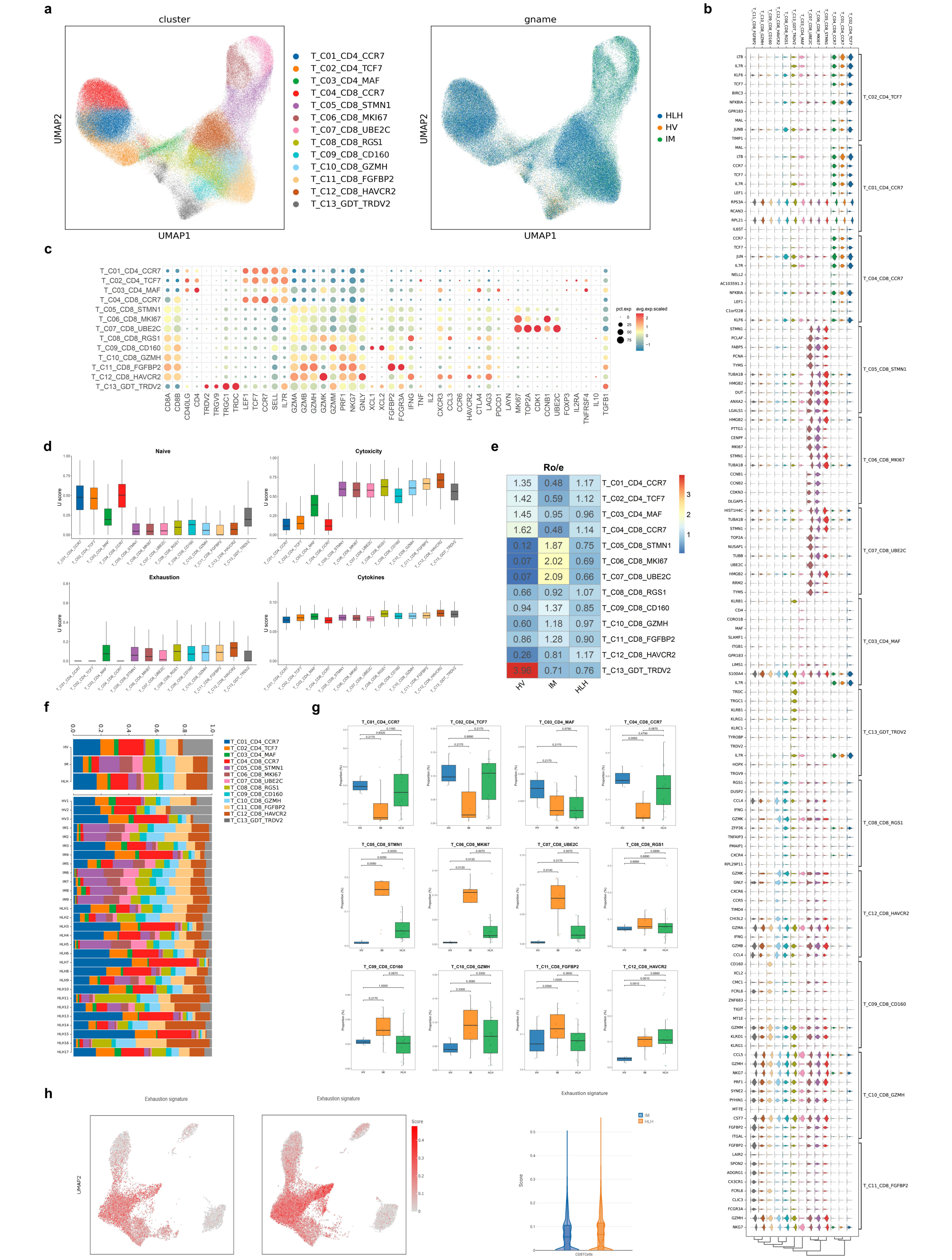

Supplementary Fig. 2: Identification of T cell clusters. (a) UMAP visualization of 165,297 single T cells from 29 samples. Cells were colored according to clusters and groups. (b) Violin plot showing the expression patterns of functional markers. Each column corresponded to specific marker genes, while rows denoted clusters, colored identically to those in a. (c) Expression distribution of canonical markers identifying individual cell clusters in the dot plot. The diameter of each dot reflected the proportion of subtype cells expressing a specific gene, while the color denotes the normalized mean expression. (d) The functional scoring of naïve, cytotoxicity, exhausted and cytokine modules in various cell clusters. (e) Quantification of cell subpopulation enrichment based on Ro/e for cellular populations. (f) Cell compositions at single group and single sample level. (g) Box plot depicting the percentage of each cell cluster, with the median indicated by a horizontal line. Two-sided Wilcoxon tests were performed between groups and *P* values were adjusted using the BH correction. (h) Comparison of exhaustion scores in T cell populations between the IM and HLH groups.

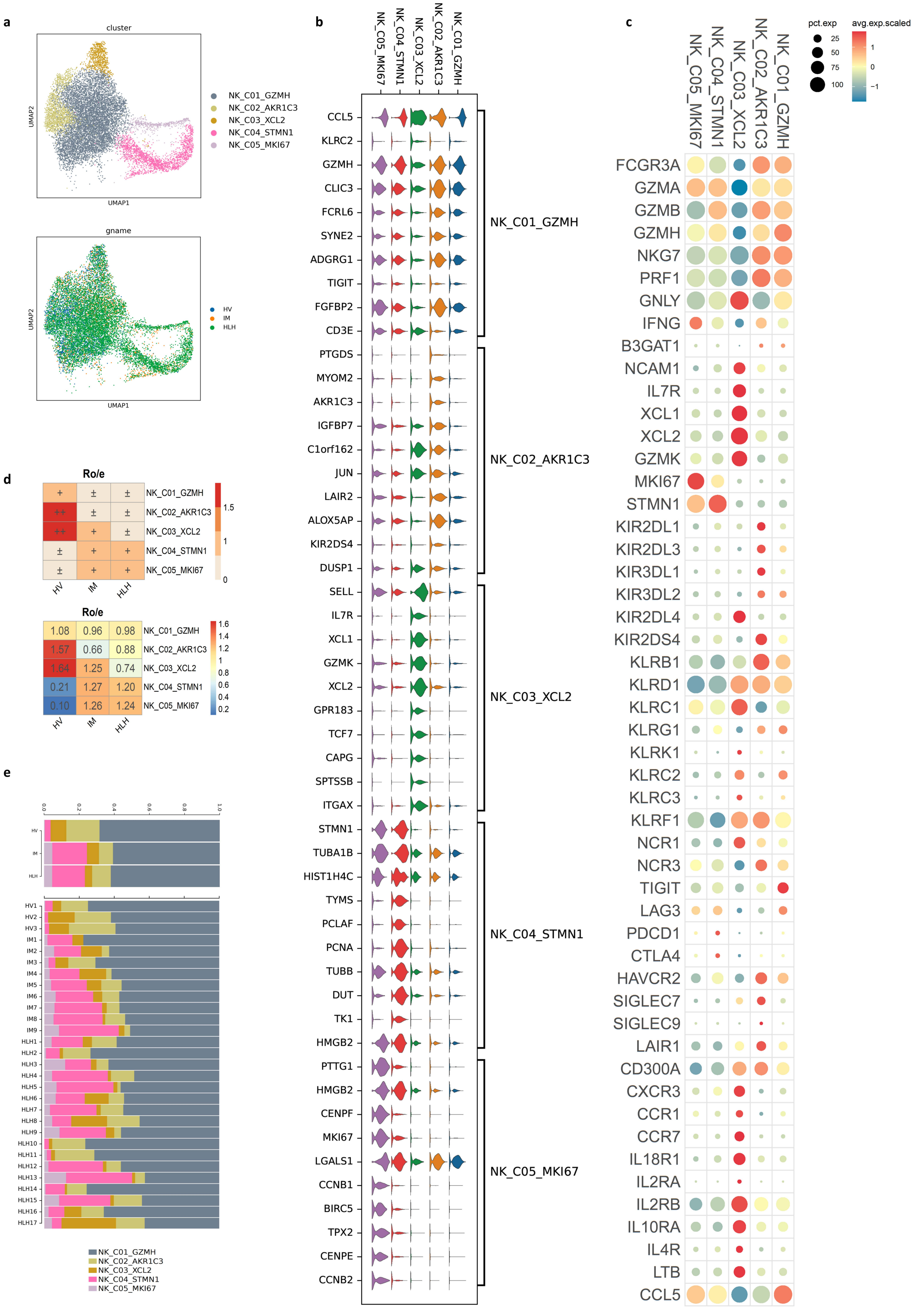

Supplementary Fig. 3: Identification of NK cell clusters. (a) Visualization through UMAP of 13,511 single NK cells derived from 29 samples. Cells were colored according to clusters and groups. (b) Violin plot showing the expression patterns of functional markers. Each column corresponded to specific marker genes, while rows denoted clusters, colored identically to those in a. (c) Expression distribution of canonical markers identifying individual cell clusters in the dot plot. The diameter of each dot reflected the proportion of subtype cells expressing a specific gene, while the color denotes the normalized mean expression. (d) Quantification of cell subpopulation enrichment based on Ro/e for cellular populations. (e) Cell compositions at single group and single sample level.

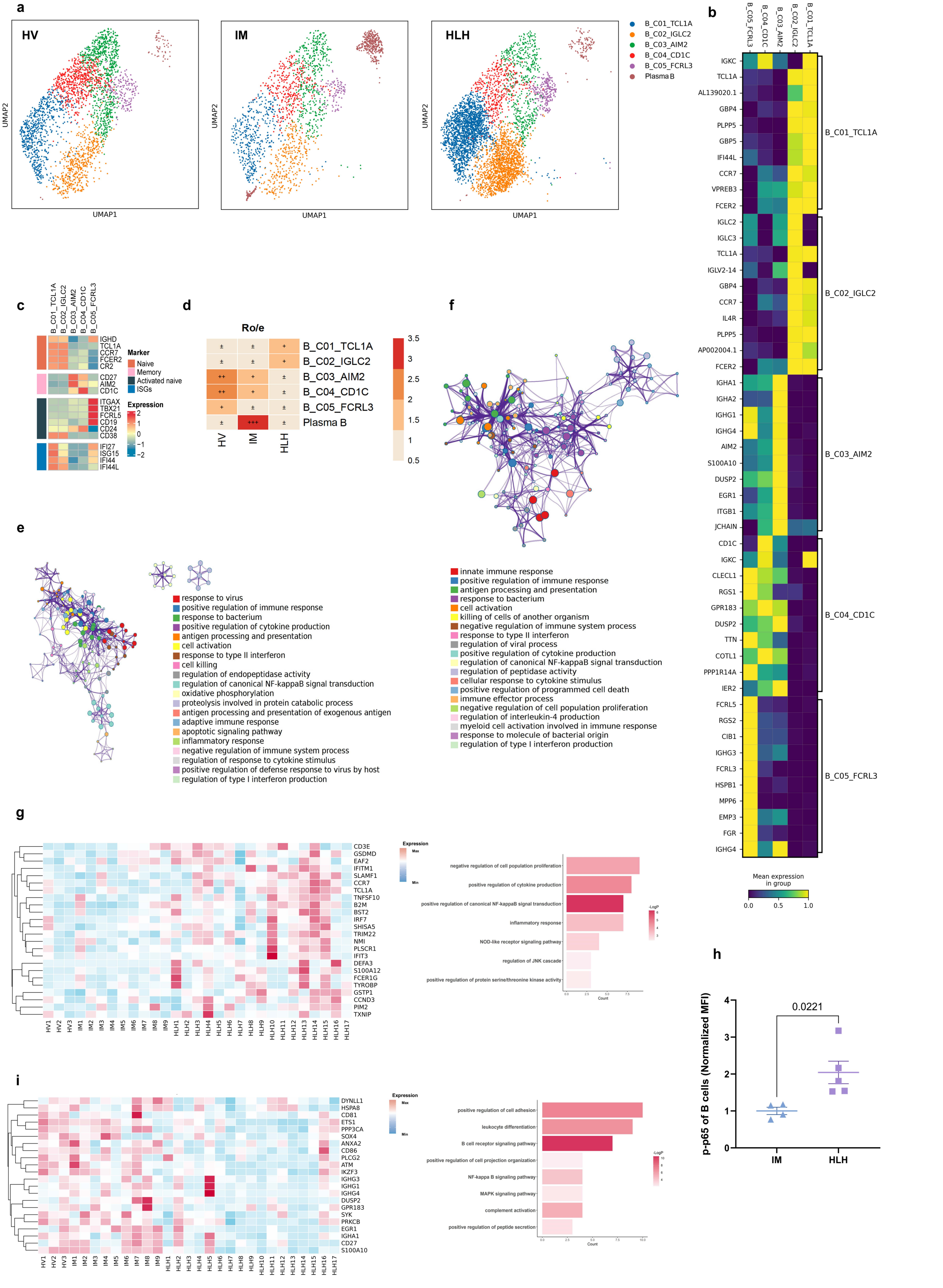

Supplementary Fig. 4: Immunological characterization of B cell clusters. (a) Visualization of 8,806 single B cells and 2,474 plasma cells from HV (n = 3), IM (n=9) and HLH (n=17) in UMAP. B cells were divided into 5 clusters. (b) Heatmap illustrating the average expression values of the top 10 highly expressed genes within each B cluster. (c) The average expression of selected markers of B cells in Heatmap. (d) Quantification of cell subpopulation enrichment based on Ro/e for cellular populations. Enriched GO terms for upregulated genes in HLH patients compared to HV (e) and IM (f). (g - h) The expression profiles of specific genes in all samples and their enrichment in the GO and KEGG pathways. Compared to the HV group and IM group, these genes were upregulated (g) or downregulated (i) in the HLH group. However, the expression levels between the HV and IM groups showed no significant changes, or the trend was inverted. The enrichment results were produced by Metascape and BH-corrected. (h) Quantification of phosphorylated p65 levels (normalized MFI values) in B cells from pediatric IM (n=4) and HLH (n=5) biologically independent samples. Two-sided Independent-samples *t* test was applied. Independent-samples *t* test was applied.

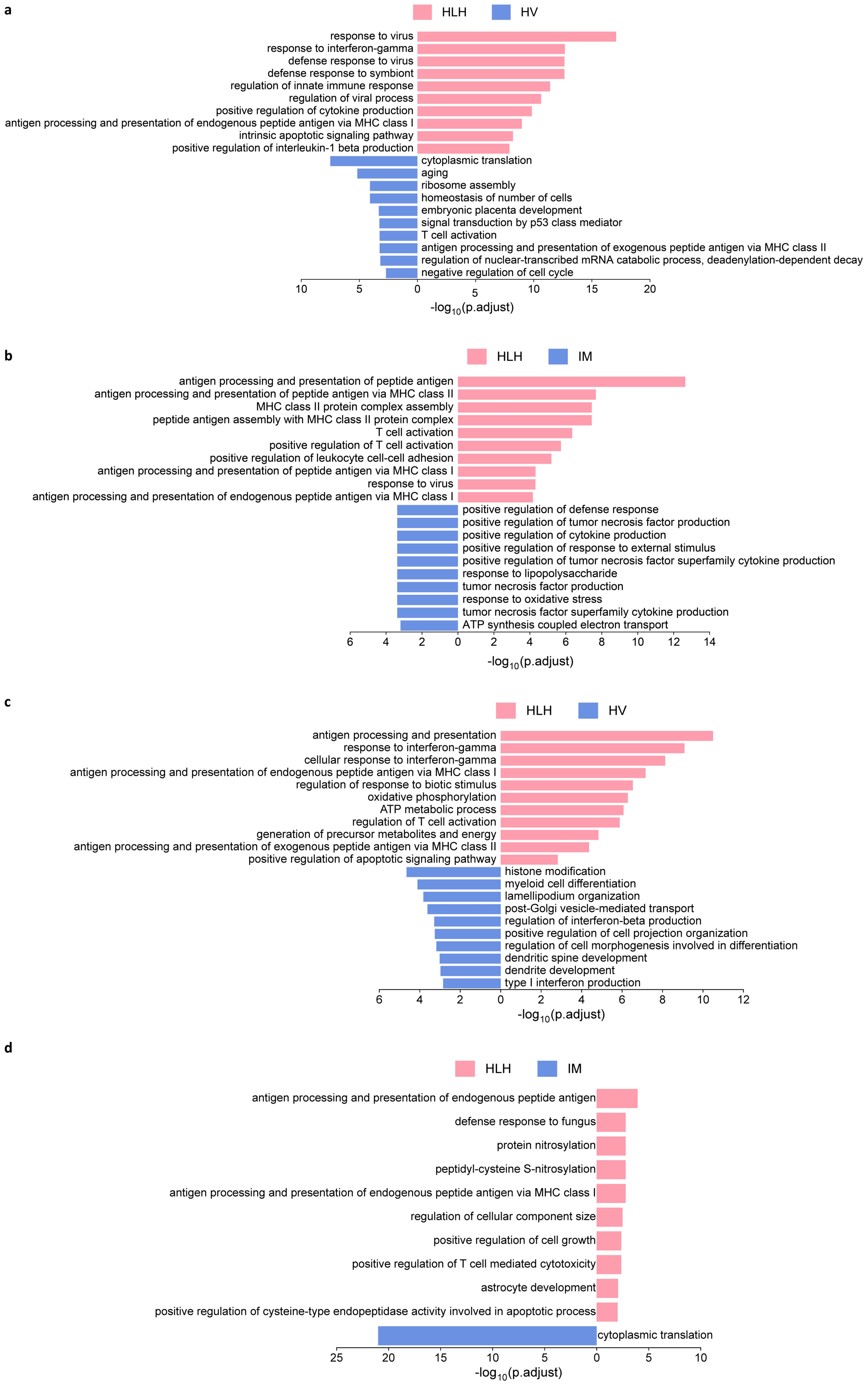

Supplementary Fig. 5: Identification of DCs clusters. (a) Enriched GO terms of differentially expressed genes in cDC between HLH and HV groups. (b) Enriched GO terms of differentially expressed genes in cDC between HLH and IM groups. (c) Enriched GO terms of differentially expressed genes in pDC between HLH and HV groups. (d) Enriched GO terms of differentially expressed genes in pDC between HLH and IM groups. The enrichment results in a – d were produced by clusterProfiler and BH-corrected.

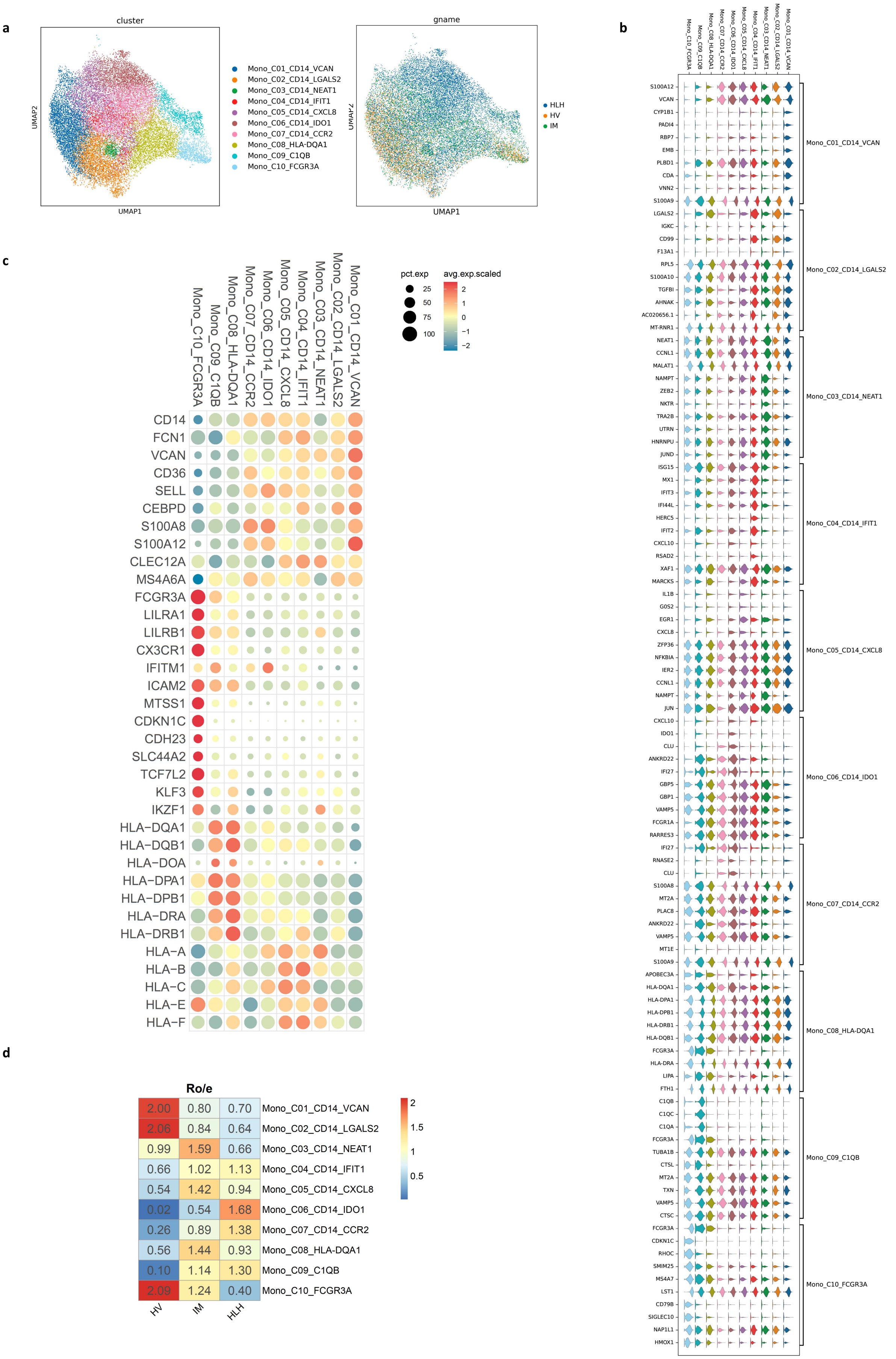

Supplementary Fig. 6: Identification of monocytes clusters. (a) UMAP visualization of 24,913 single monocytes from 29 samples. Cells were colored according to clusters and groups. (b) Violin plot showing the expression patterns of functional markers. Each column corresponded to specific marker genes, while rows denoted clusters, colored identically to those in a. (c) Expression distribution of canonical markers identifying individual cell clusters in the dot plot. The diameter of each dot reflected the proportion of subtype cells expressing a specific gene, while the color denotes the normalized mean expression. (d) Quantification of cell subpopulation enrichment based on Ro/e for cellular populations.

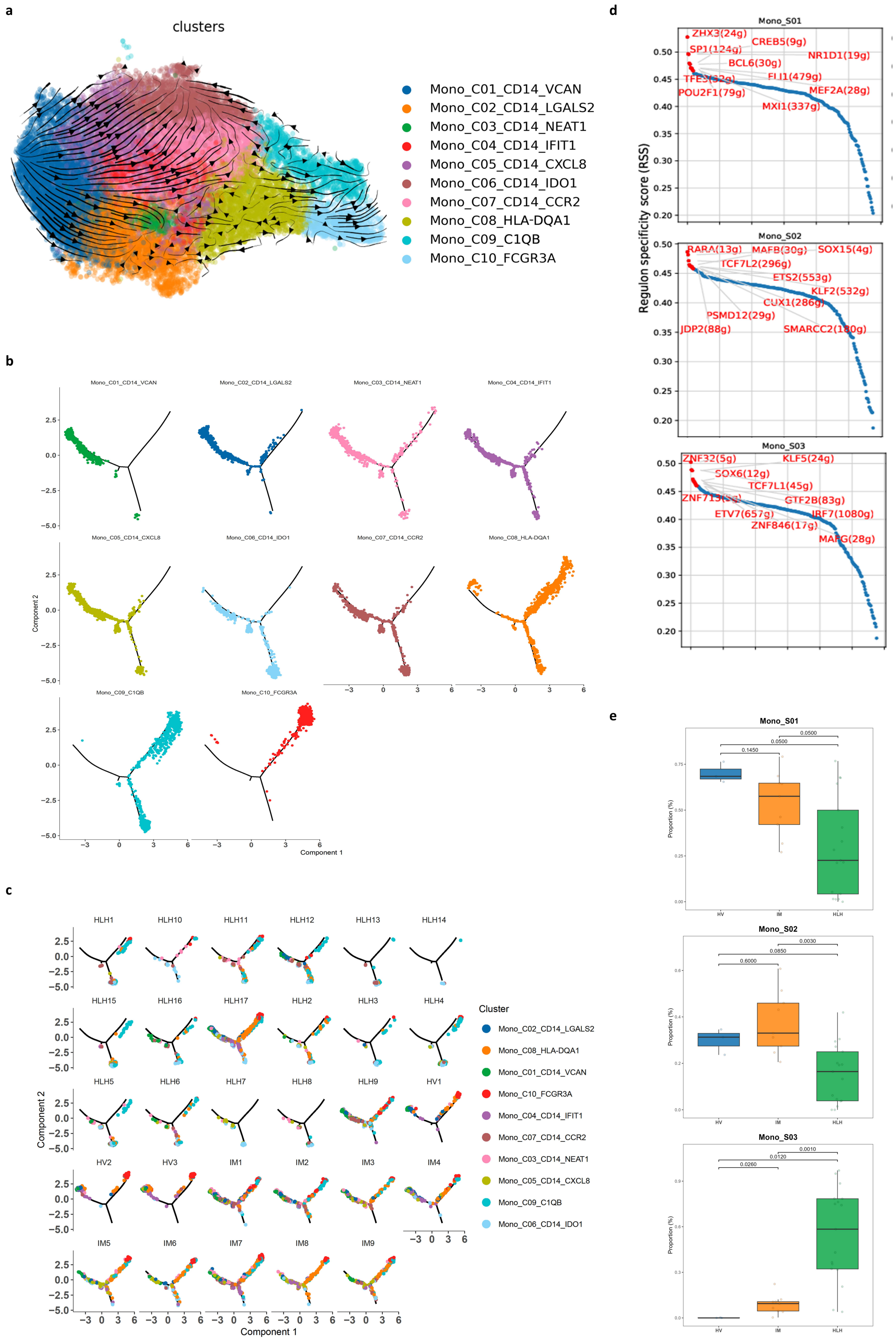

Supplementary Fig. 7: Trajectory comparisons among all monocyte clusters. (a) RNA velocity to transient cell states. Arrows indicated the direction of differentiation. Pseudotime trajectory analysis of monocytes among each cell cluster (b) and sample (c), where each dot signified an individual cell and was colored according to its cluster label. (d) The top 10 transcriptional regulons in each cell type. (e) Box plot depicting the percentage of each cell cluster in HV (n=3), IM (n=9) and HLH (n=17) group, with the median indicated by a horizontal line. Two-sided Wilcoxon tests were performed between groups and *P* values were adjusted using the BH correction.

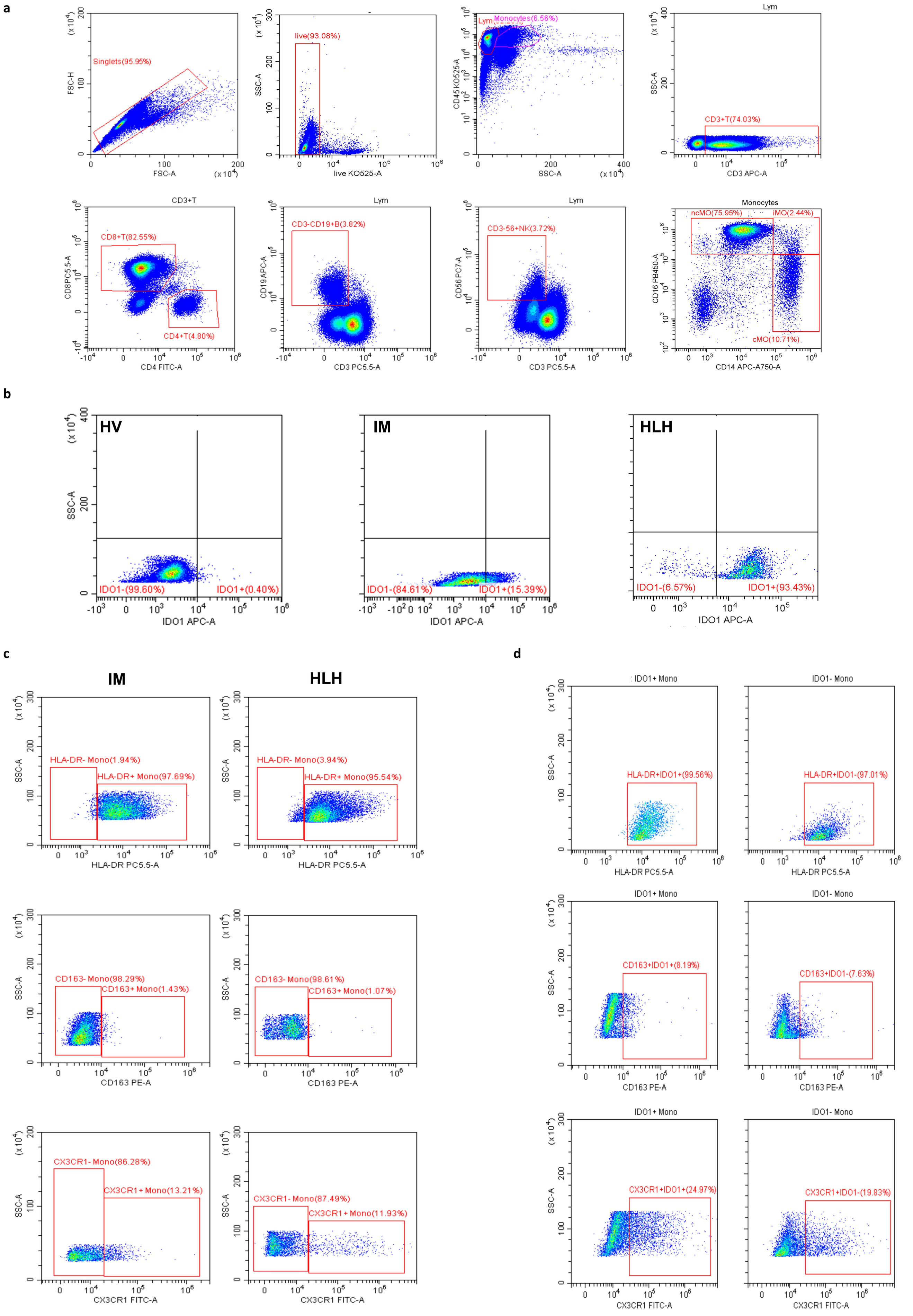

Supplementary Fig. 8: Flow gating strategy and FACS plots for staining. (a) Flow gating strategy for peripheral blood samples containing T, B, NK and monocytes. (b) FACS plots of IDO1 staining of monocyte populations in the HV, IM and HLH groups. (c) FACS plots of HLA-DR, CD163 and CX3CR1 staining of monocyte populations in the IM and HLH groups. (d) In monocyte subsets of HLH patients, FACS plots of HLA-DR^{+}/CD163^{+}/CX3CR1^{+} proportions between IDO1^{+} and IDO1^{-} subpopulations.

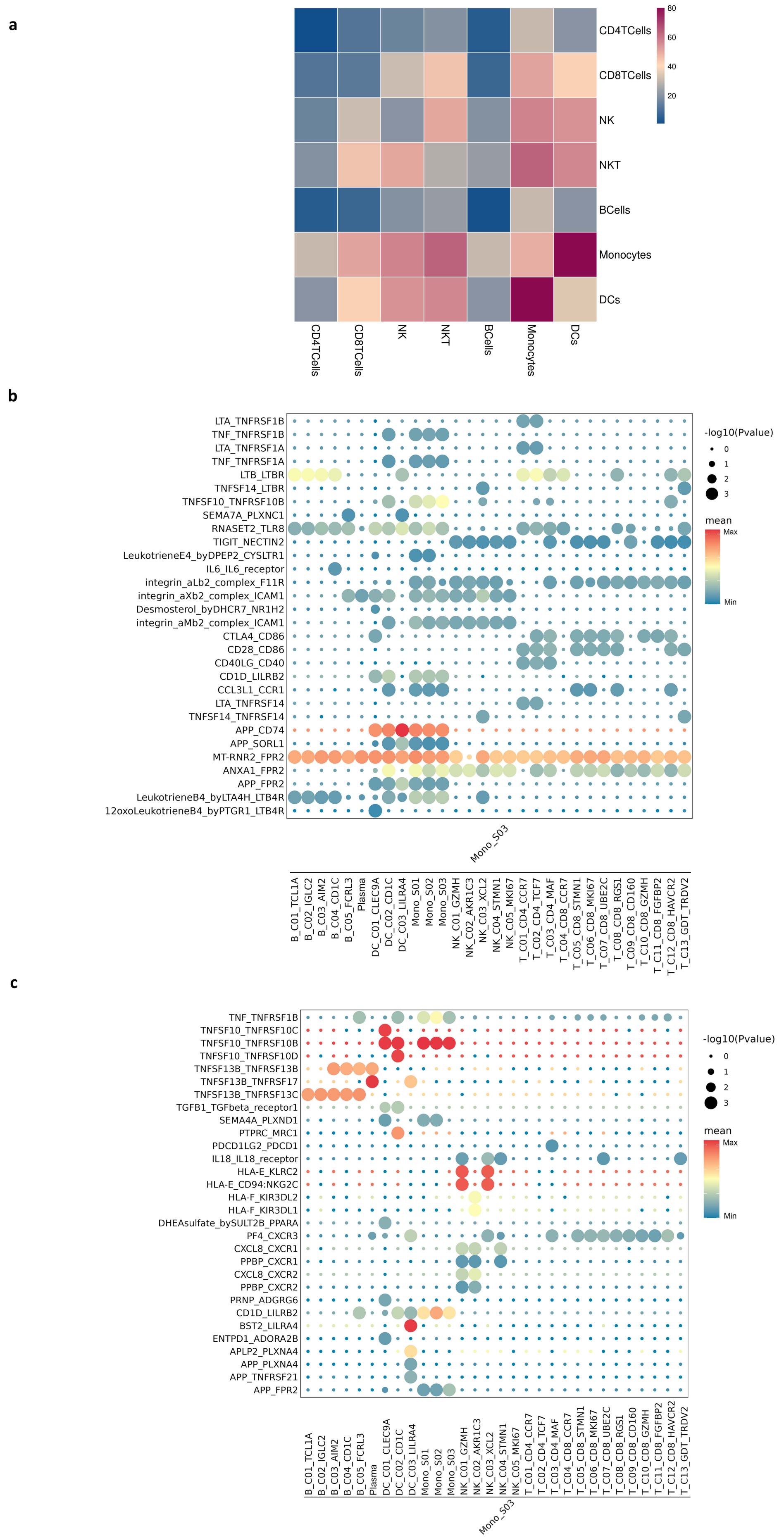

Supplementary Fig. 9: Analysis of cellular communication within the HLH group. (a) Heatmap depicting the quantity of interaction pairs between two cell types, where intensification of the color gradient signified an increased count of interaction events. Bubble chart depicting the top 30 predicted interaction pairs involving Mono-S03 as the receptor (b) and ligand (c) cells with various other cell types. The circle size in this illustration reflected the significance *P* value of the ligand-receptor axis, and the color indicated the specificity of the interactions. The interaction results were produced by CellPhoneDB.

Supplementary Table 1: Baseline characteristics of scRNA-seq group

	HV	IM	HLH
Age category — no.			
<2	0	1	1
[2-6)	2	3	13
[6-12)	1	5	3
[12-18]	0	0	0
Median age (range) — yr	5.3 (4.9 - 6.3)	6.0 (1.5-11.0)	4.1 (1.7-10)
EBV-DNA copy (whole blood) — no.			
＜1000	1	0	0
1000-10000	2	4	2
>10000	-	5	15
Median EBV-DNA copy(range) — copies/mL	1,310 (634 - 2,410)	15,000 (1,050 - 102,100)	384,000 (1,730 – 9,500,000)
Serum ferritin — no.			
＜500	-	2	1
500-5000	-	1	8
5001-10000	-	-	1
>10000	-	-	7
unknown	3	6	0
Median Serum ferritin (range) —μg/L	-	156 (114 - 3,707)	4,045 (309 - 40,000)
sCD25— no.			
＜2400	-	-	0
2400-5000	-	-	1
>5000	-	-	16
Median sCD25(range) —U/mL	-	-	38,360 (4,107 - 227,675)
Efficacy — no.	-		
complete response, CR	-	-	15
partial response, PR	-	-	0
no response, NR	-	-	2
Outcome — no.			
alive	-	9	15
deceased	-	0	2

Supplementary Table 2: Clinical characteristics of scRNA-seq group

Sample	Gender	Race	Age range (years)	Interval between sampling and admission (days)	Treatment	Therapeutic Course (weeks)	Response Evaluation	Clinical Outcomes	EBV-DNA	EBV-infected cell type	EBV-VCA-IgM	EBV-VCA-IgG	EBV-EA-IgG	EBNA-IgG	Hepatomegaly	Splenomegaly	Lymphadenopathy	Marrow haemophagocyte
reference (normal) ranges									< 400 (copies/mL)		(-)	(-)	(-)	(-)				
HLH1	Female	East Asian (China)	(0-6)	1	HLH-1994	8	CR	alive	9.29E+05	B, NK, T	(+)	(+)	(-)	(-)	Yes	Yes	No	Yes
HLH2	Male	East Asian (China)	(0-6)	2	HLH-1994	8	CR	alive	1.73E+03	B	(+)	(-)	(+)	(-)	Yes	No	No	Yes
HLH3	Male	East Asian (China)	(0-6)	1	HLH-1994	8	CR	alive	3.84E+05	B, NK, T	(+)	(-)	(+)	(-)	Yes	No	Yes	Yes
HLH4	Male	East Asian (China)	(0-6)	2	HLH-1994	8	CR	alive	1.14E+06	B, NK, T	(+)	(+)	(+)	(-)	Yes	Yes	No	Yes
HLH5	Male	East Asian (China)	[6-12)	1	HLH-1994	8	CR	alive	3.08E+04	B, NK	(+)	(+)	(-)	(-)	Yes	Yes	No	Yes
HLH6	Female	East Asian (China)	[6-12)	0	HLH-1994	2	CR	alive	9.46E+03	B, NK	(+)	(+)	(-)	(-)	Yes	Yes	No	Yes
HLH7	Female	East Asian (China)	[6-12)	0	HLH-1994	0 (1 day)	NR	deceased	5.04E+06	B, NK, T	(+)	(+)	(+)	(-)	Yes	Yes	Yes	Yes
HLH8	Female	East Asian (China)	[6-12)	2	HLH-1994	8	CR	alive	2.24E+04	B, NK, T	(+)	(+)	(+)	(-)	Yes	Yes	No	Yes
HLH9	Female	East Asian (China)	(0-6)	2	HLH-1994	2	CR	alive	3.40E+04	B, NK, T	(+)	(+)	(-)	(-)	Yes	Yes	No	Yes
HLH10	Male	East Asian (China)	(0-6)	2	HLH-1994	8	CR	alive	9.50E+06	B, NK, T	(+)	(+)	(+)	(-)	Yes	Yes	No	Yes
HLH11	Female	East Asian (China)	(0-6)	3	HLH-1994	8	CR	alive	2.87E+06	B	(+)	(+)	(+)	(-)	Yes	Yes	Yes	Yes
HLH12	Female	East Asian (China)	(0-6)	3	HLH-1994	2	CR	alive	1.50E+05	B, NK, T	(+)	(-)	(-)	(-)	Yes	Yes	No	No
HLH13	Male	East Asian (China)	(0-6)	2	HLH-1994	8	CR	alive	1.48E+06	B, NK, T	(+)	(+)	(-)	(-)	No	No	No	Yes
HLH14	Female	East Asian (China)	(0-6)	1	HLH-1994	8	CR	alive	2.59E+04	B, NK	(+)	(+)	(-)	(-)	Yes	No	No	Yes
HLH15	Male	East Asian (China)	(0-6)	2	HLH-1994	8	CR	alive	1.07E+06	B, NK, T	(+)	(+)	(+)	(-)	Yes	Yes	No	Yes
HLH16	Female	East Asian (China)	(0-6)	1	HLH-1994	2	NR	deceased	2.03E+06	B, NK	(+)	(+)	(-)	(-)	Yes	Yes	No	No
HLH17	Female	East Asian (China)	(0-6)	2	HLH-1994	8	CR	alive	2.75E+05	B, NK, T	(+)	(+)	(-)	(-)	Yes	Yes	No	Yes
IM1	Male	East Asian (China)	(0-6)	1	Symptomatic treatment (without hormones or chemotherapy)	1	CR	alive	1.50E+04	NA	(+)	(-)	(-)	(-)	Yes	Yes	Yes	NA
IM2	Male	East Asian (China)	[6-12)	1	Symptomatic treatment (without hormones or chemotherapy)	1	CR	alive	1.02E+05	NA	(+)	(-)	(-)	(-)	Yes	No	Yes	NA
IM3	Male	East Asian (China)	(0-6)	2	Symptomatic treatment (without hormones or chemotherapy)	1	CR	alive	2.99E+04	NA	(+)	(+)	(-)	(-)	No	No	Yes	NA
IM4	Male	East Asian (China)	(0-6)	0	Symptomatic treatment (without hormones or chemotherapy)	1	CR	alive	4.03E+03	NA	(+)	(+)	(-)	(-)	No	No	Yes	NA
IM5	Female	East Asian (China)	(0-6)	1	Symptomatic treatment (without hormones or chemotherapy)	1	CR	alive	5.00E+03	NA	(+)	(+)	(-)	(-)	Yes	Yes	Yes	NA
IM6	Female	East Asian (China)	[6-12)	1	Symptomatic treatment (without hormones or chemotherapy)	1	CR	alive	7.98E+03	NA	(+)	(+)	(-)	(-)	Yes	No	Yes	NA
IM7	Male	East Asian (China)	[6-12)	0	Symptomatic treatment (without hormones or chemotherapy)	1	CR	alive	2.21E+04	NA	(+)	(+)	(+)	(-)	Yes	Yes	Yes	NA
IM8	Female	East Asian (China)	[6-12)	1	Symptomatic treatment (without hormones or chemotherapy)	1	CR	alive	3.35E+04	NA	(+)	(+)	(-)	(-)	Yes	Yes	Yes	NA
IM9	Male	East Asian (China)	[6-12)	1	Symptomatic treatment (without hormones or chemotherapy)	1	CR	alive	1.05E+03	NA	(+)	(+)	(-)	(-)	Yes	Yes	Yes	NA
HV1	Female	East Asian (China)	(0-6)	0	NA	NA	NA	alive	1.31E+03	NA	NA	NA	NA	NA	No	No	No	NA
HV2	Male	East Asian (China)	(0-6)	0	NA	NA	NA	alive	6.34E+02	NA	NA	NA	NA	NA	No	No	No	NA
HV3	Male	East Asian (China)	[6-12)	0	NA	NA	NA	alive	2.41E+03	NA	NA	NA	NA	NA	No	No	No	NA

Sample	Serum ferritsn	Natural killer cell function assay	sCD25	White blood cell count	Absolute lymphocyte count	Absolute monocyte count	Absolute neutrophil count	Red blood cell	Hemoglobin	Platelet count	Triglyceride	Aspartate aminotransferase	Alanine Transaminase	Lactate dehydrogenase	Total Protein	Albumin	Total Bilirubin	Direct Bilirubin	APTT	PT	TT	FIB
reference (normal) ranges	20 - 110 (μg/L)	> 15%	< 2400 (U/mL)	3.5-9.5 (10^9/L)	1.1-3.2 (10^9/L)	0.1-0.6 (10^9/L)	1.8-6.3 (10^9/L)	3.8-5.1 (10^{12}/L)	115-150 (g/L)	125-350 (109/L)	< 1.7 (mM)	13-15 (U/L)	7-40 (U/L)	120-250 (IU/L)	65-85 (g/L)	40-55 (g/L)	3.42-20.5 (μM)	0-6.84 (μM)	20-40 (s)	9-14 (s)	14-21 (s)	2-4 (g/L)
HLH1	20000	1.35%	44020	0.89	0.39	0.02	0.48	1.76	45	25	4.46	343	104	1,752	41.9	24.4	6.1	3.4	45.2	12.9	25.4	0.77
HLH2	8866	3.85%	4107	9.72	2.1574	0.165624	0.348936	1.81	106.7	179.5	3.75	1478	1011	1856	73.1	39.0	52.1	46.8	50.9	12.0	11.6	2.26
HLH3	4045	15.03%	227675	1.06	0.88	0.06	0.15	2.97	91	57	2.76	143	83	1221	53.7	32.4	8.8	3.5	46.2	12.4	22.5	1.18
HLH4	2740	19.07%	44000	0.41	0.17	0.06	0.36	3.43	107	61	2.65	51	31	478	50.1	27.4	6.5	0.0	25.2	11.7	21.6	0.91
HLH5	1650	33.67%	39977	11.25	7.76	0.23	3.03	3.67	90	120	3.62	565.2	81.4	3116.3	42.7	23.4	55.6	37.7	34.1	13.1	21.7	0.89
HLH6	1232	10.60%	13680	1.87	1.28	0.15	0.44	3.33	83.9	44	2.64	310	126	726	61.7	30.9	10.5	5.3	42.6	12.3	24.4	1.15
HLH7	1983	8.11%	19760	1.95	1.47	0.08	0.39	4.18	108.2	21	9.46	876	384	4389	40.3	21.0	147.3	132.0	49.8	16.7	28.6	0.73
HLH8	38851	19.51%	44000	1.52	0.96	0.05392	0.5	3.56	107	71	4.59	555	1177	616	45.0	25.2	47.7	45.1	24.5	11.3	22.1	0.92
HLH9	2297	13.36%	9499	0.66	0.56	0.02	0.07	3.41	92.2	38	3.8	353	118	2840	54.2	31.6	64.0	59.7	22.1	11.5	21.9	1.03
HLH10	309	19.00%	33742	2.58	1.49	0.03226	1.05	2.81	86	84	4.02	310	162	680	84.0	34.9	11.5	8.7	30.3	11.2	21.1	1.10
HLH11	947	35.38%	85750	15.12	13.71	0.28	1.09	3.68	103	40	3.77	96	82	668	68.6	33.6	10.2	5.3	30.1	12.3	12.3	1.22
HLH12	13644	29.97%	33743	3.37	2.28	0.12	0.96	4.07	111.0	46	3.50	381	108	799	61.7	28.7	14.20	12.80	32.30	10.70	21.2	2.30
HLH13	40000	17.13%	8680	1.48	0.47	0.12	0.88	2.57	68	74.7	3.39	350	262	2269	46.9	30.0	74.1	56.5	37	11.8	14.8	1.26
HLH14	1650	19.37%	19943	2.04	1.63	0.26	0.14	2.88	67	34	2.23	119	93	1144	55.1	33.5	3.2	2.3	35.6	12.8	23.3	1.04
HLH15	19852	14.08%	38360	0.64	0.41	0.0368	0.19	3.05	89	62	3.37	641	467	2880	42.3	22.1	15.4	0.0	44.3	14.5	23.2	0.97
HLH16	31470	0.25%	40467	6.94	4.26	0.11	2.49	3.22	85	17	3.86	399	135.4	2403.3	57.3	36.2	10.8	2.4	39.4	11.8	34.4	0.87
HLH17	26503	16.23%	40514	3.18	1.95	0.2	0.95	2.87	73.7	46.4	5.60	358	285	1605	44.4	23.1	109.1	91.2	42.7	14.4	16.9	0.95
IM1	NA	NA	NA	10.88	4.46	1.05	5.33	4.89	130	560	0.98	77	35	390	60.3	40.2	5.1	2.7	NA	NA	NA	NA
IM2	114	NA	NA	14.08	7.30	1.11	5.41	4.67	131	225	1.76	34	17	462	71.3	42.3	5.7	2.2	27.9	13.1	16.8	2.87
IM3	NA	NA	NA	14.92	7.06	1.82	6.01	4.48	122	188	1.24	64	71	552	67.6	38.2	4.4	1.4	NA	NA	NA	NA
IM4	NA	NA	NA	10.71	4.82	0.92	4.9	4.81	122	347	0.69	135	218	352	70.4	41.4	6	2.9	25.8	12.8	16.9	2.15
IM5	NA	NA	NA	9.11	5.17	0.64	2.87	4.02	111	349	1.08	70	117	532	69.2	41.7	4.4	1.6	NA	NA	NA	NA
IM6	NA	NA	NA	14.84	13.03	0.33	1.22	4.05	107	217	1.03	83	205	526	68.4	38.2	4.5	1.8	25.8	11.6	17.5	2.82
IM7	156	NA	NA	11.19	5.99	1.22	3.95	4.18	125	220	1.10	93	196	442	63.3	37.9	6.7	2.1	28.7	12.8	18.2	3.03
IM8	NA	NA	NA	13.6	8.17	1.60	3.81	4.47	126	122	1.61	116	115	604	80.4	35.4	5.4	2.4	NA	NA	NA	NA
IM9	3707	NA	NA	14.16	7.45	0.65	6.05	4.57	122	286	1.43	233	268	506	61.1	34.2	5.2	2.3	NA	NA	NA	NA
HV1	NA	NA	NA	6.82	2.24	0.42	3.55	3.96	131	311	NA	27	15	NA	70.2	45.4	10.9	1.9	NA	NA	NA	NA
HV2	NA	NA	NA	4.94	2.39	0.41	1.87	4.13	115	244	NA	24	11	NA	67.7	44.9	9.5	2.9	NA	NA	NA	NA
HV3	NA	NA	NA	5.98	1.99	0.74	3.14	4.97	125	332	NA	27	16	NA	74.1	48.7	9.2	1.8	NA	NA	NA	NA

NA：Not Applicable; CR: Complete Response; NR: No Response

Supplementary Table 3: Panel of excluded gene mutations associated with HLH and primary immunodeficiency diseases based on whole-exome sequencing results

Gene lists
ADA, AK2, AP3B1, ARHGAP21, ATM, BLM, BLOC1S6, BTK, CADPS2, CARD11, CASP10, CASP8, CCDC141, CD127, CD27, CD3E, CD40LG, CD70, CDC42, COG1, COG6, CORO1A, CTPS1, CYBB, DCLRE1C, DKC1, DNMT3B, DOCK8, EXPH5, FADD, FAM160A2, FAS, FASLG, FKBPL, GATA2, GDI1, IFNGR1, IFNGR2, IKBKG, IL10, IL21R, IL27RA, IL2RA, IL2RG, IRF5, IRF8, ITK, JAK3, KIR2DS5, KIR3DS1, LRBA, LRGUK, LYST, MAGT1, MCM4, MEFV, MICAL2, MVK, NCF2, NCF4, NLRC4, NLRP12, NLRP3, OSTM1, PIK3CD, PIK3R1, PLCG2, PNP, PRF1, PRKCD, RAB27A, RAG1, RAG2, RASGRP1, RECQL4, SH2D1A, SH3BP2, SLC29A3, SLC7A7, STAT1, STX11, STXBP2, TCIRG1, TGFB, TNFRSF11A, TNFRSF13B, TNFRSF1A, UNC13D, UNG, WAS, XIAP, XIRP2, ZAP70

Supplementary Table 4: Genes related to functional module scores

Categories	Gene lists
Inflammatory	*ABCA1, ABI1, ACVR1B, ACVR2A, ADGRE1, ADM, ADORA2B, ADRM1, AHR, APLNR, AQP9, ATP2A2, ATP2B1, ATP2C1, AXL, BDKRB1, BEST1, BST2, BTG2, C3AR1, C5AR1, CALCRL, CCL17, CCL2, CCL20, CCL22, CCL24, CCL5, CCL7, CCR7, CCRL2, CD14, CD40, CD48, CD55, CD69, CD70, CD82, CDKN1A, CHST2, CLEC5A, CMKLR1, CSF1, CSF3, CSF3R, CX3CL1, CXCL10, CXCL11, CXCL6, CXCL8, CXCL9, CXCR6, CYBB, DCBLD2, EBI3, EDN1, EIF2AK2, EMP3, EREG, F3, FFAR2, FPR1, FZD5, GABBR1, GCH1, GNA15, GNAI3, GP1BA, GPC3, GPR132, GPR183, HAS2, HBEGF, HIF1A, HPN, HRH1, ICAM1, ICAM4, ICOSLG, IFITM1, IFNAR1, IFNGR2, IL10, IL10RA, IL12B, IL15, IL15RA, IL18, IL18R1, IL18RAP, IL1A, IL1B, IL1R1, IL2RB, IL4R, IL6, IL7R, INHBA, IRAK2, IRF1, IRF7, ITGA5, ITGB3, ITGB8, KCNA3, KCNJ2, KCNMB2, KIF1B, KLF6, LAMP3, LCK, LCP2, LDLR, LIF, LPAR1, LTA, LY6E, LYN, MARCO, MEFV, MEP1A, MET, MMP14, MSR1, MXD1, MYC, NAMPT, NDP, NFKB1, NFKBIA, NLRP3, NMI, NMUR1, NOD2, NPFFR2, OLR1, OPRK1, OSM, OSMR, P2RX4, P2RX7, P2RY2, PCDH7, PDE4B, PDPN, PIK3R5, PLAUR, PROK2, PSEN1, PTAFR, PTGER2, PTGER4, PTGIR, PTPRE, PVR, RAF1, RASGRP1, RELA, RGS1, RGS16, RHOG, RIPK2, RNF144B, ROS1, RTP4, SCARF1, SCN1B, SELE, SELENOS, SELL, SEMA4D, SERPINE1, SGMS2, SLAMF1, SLC11A2, SLC1A2, SLC28A2, SLC31A1, SLC31A2, SLC4A4, SLC7A1, SLC7A2, SPHK1, SRI, STAB1, TACR1, TACR3, TAPBP, TIMP1, TLR1, TLR2, TLR3, TNFAIP6, TNFRSF1B, TNFRSF9, TNFSF10, TNFSF15, TNFSF9, TPBG, VIP*
Cytokines	*IL2, IL7, CSF3, CXCL10, CCL2, CCL3, TNF, TFTN1, IL6, CCL7, IL1RN, CSF1, IFNG, IL2RA, IL10, IL18, HGF, CXCL9, CCL27, TGFB1, IL1B, LTA, CSF2, LTB, TNFSF13, IL4, CCL12, CXCL8, CXCL11, CCL4, CXCL1, CXCL2, CXCL3, CCL3L1, CCL8, CXCL16, CCL5, CCL11, IFNA2, IFNA1, CCL20, CCL4L2, OSM, TNFSF14, SA100A12, FGF19, CXCL5, CCL19, IL18R1, TGFA, IFNB1, IL8, IL17C, TNFSF10, FGF7, XCL1, FGF13, LIF, TGFB3, INHBE, CERS1, TXLNA, IFNW1, IL22, XCL2, CCL25, CCL16, CD40LG, IL20, FASLG, TPO, SCYL3, PF4V1, TNFSF8, GDF15, IL1A, VEGFA, GDF7, BMP6, PDGFA, IL21, ABCD-1, ABCD-2, PDGFB, TNFSF4, FAM19A1, HBEGF, PDGFD, IL12RB2, GH1, VEGFB, MIP3B, IL27, PF4, BMP8B, TNFSF12, IL15, SCYL2, SCYL1, TSLP, GDF11, SDF1B, INHBA, PPBP, FGF11, IFNG-AS1, FGF22, VEGFC, CCL18, TNFSF11, IL12A, EBI3, AMH, IL26, IL32, PDGFC, FGF23, IGF1, IL1F11, CCL28, CLCF1, TNFSF9, BMP3, IL24, GDF10, CXCL6, GDF9, IL23A, IL16, CD70, IL5, FGF9, IFNL1, TSC1, FGF2, IL23R, IL1G, SPP1, IL12RB1, BMP4, IL13, TPAR1, TGFB2, FAM19A2, AGIF3, EDA, MIF, TNFSF13B, BMP7, FGF18, CCL23, IL12B, IL17A, CD163, IL33*
Naïve	*CCR7, TCF7, LEF1, SELL*
Cytotoxicity	*PRF1, IFNG, GNLY, NKG7, GZMB, GZMA, GZMH, KLRK1, KLRB1, KLRD1, CTSW, CST7*
Exhausted	*LAG3, TIGIT, PDCD1, CTLA4, HAVCR2*

Supplementary Table 5: qRT-PCR primer sequences

Primer Name	Forward primer sequences (5’- 3’)	Reverse primer sequences (5’- 3’)
IL1A	TGTATGTGACTGCCCAAGATGAAG	AGAGGAGGTTGGTCTCACTACC
IL1B	CCACAGACCTTCCAGGAGAATG	GTGCAGTTCAGTGATCGTACAGG
IL6	AGACAGCCACTCACCTCTTCAG	TTCTGCCAGTGCCTCTTTGCTG
CXCL8	GAGAGTGATTGAGAGTGGACCAC	CACAACCCTCTGCACCCAGTTT
IL10	TCTCCGAGATGCCTTCAGCAGA	TCAGACAAGGCTTGGCAACCCA
IL12A	TGCCTTCACCACTCCCAAAACC	CAATCTCTTCAGAAGTGCAAGGG
IL17A	CGGACTGTGATGGTCAACCTGA	GCACTTTGCCTCCCAGATCACA
IL18	GATAGCCAGCCTAGAGGTATGG	CCTTGATGTTATCAGGAGGATTCA
CSF2	GGAGCATGTGAATGCCATCCAG	CTGGAGGTCAAACATTTCTGAGAT
CCL2	AGAATCACCAGCAGCAAGTGTCC	TCCTGAACCCACTTCTGCTTGG
CCL3	ACTTTGAGACGAGCAGCCAGTG	TTTCTGGACCCACTCCTCACTG
CCL4	GCTTCCTCGCAACTTTGTGGTAG	GGTCATACACGTACTCCTGGAC
CCL5	CCTGCTGCTTTGCCTACATTGC	ACACACTTGGCGGTTCTTTCGG
CCL7	ACAGAAGGACCACCAGTAGCCA	GGTGCTTCATAAAGTCCTGGACC
TNF	CTCTTCTGCCTGCTGCACTTTG	ATGGGCTACAGGCTTGTCACTC
LTA	ACACCTTCAGCTGCCCAGACTG	TCCGTGTTTGCTCTCCAGAGCA
TGFB1	TACCTGAACCCGTGTTGCTCTC	GTTGCTGAGGTATCGCCAGGAA
PTGS2	CGGTGAAACTCTGGCTAGACAG	GCAAACCGTAGATGCTCAGGGA
IFNG	GAGTGTGGAGACCATCAAGGAAG	TGCTTTGCGTTGGACATTCAAGTC
IL2RA	GAGACTTCCTGCCTCGTCACAA	GATCAGCAGGAAAACACAGCCG

Supplementary Table 6: Antibody for flow cytometry

Antidody	Supplier name	Catalog number	Dilutions
BV510 Mouse Anti-Human CD45	BD	563204	1 : 50
APC/Cyanine7 anti-human CD14	BioLegend	325619	1 : 50
BV421 Mouse Anti-Human CD16	BD	562874	1 : 100
PERCP Anti-Human HLA-DR	BioLegend	307628	1 : 100
PE Mouse Anti-Human CD163	BD	560933	1 : 100
BB515 Rat Anti-Human CX3CR1	BD	565902	1: 100
AF647 Mouse Anti-Human IDO1	BD	566648	1 : 100
APC anti-human CD3	Biolegend	317318	1 : 25
FITC Mouse Anti-Human CD4	BD	555346	1 : 8
PerCP/Cyanine5.5 anti-human CD8	Biolegend	344710	1 : 20
PerCP/Cyanine5.5 anti-human CD3	Biolegend	317336	1 : 20
APC anti-human CD19	Biolegend	302212	1 : 20
RB780 Mouse Anti-Human CD56	BD	568763	1 : 20
Alexa Fluor 700 Mouse anti-Human CD14	BD	557923	1 : 25
BV421 Mouse Anti-Human CD16	BD	562874	1 : 25
PE Mouse anti-NF-κB p65 (pS529)	BD	558423	1 : 10
