# Supplementary material for: Biomarkers of pediatric Epstein-Barr virus-associated hemophagocytic lymphohistiocytosis through single-cell transcriptomics
Source: Nat Commun. 2025 Jul 25;16:6888. doi: 10.1038/s41467-025-62090-5 (PMC12297430; doi:10.1038/s41467-025-62090-5)
Supplement: Supplementary file 2 — Reporting Summary [file 41467_2025_62090_MOESM2_ESM.pdf]

nature portfolio

Corresponding author(s): Minghua Yang, Dao Wang (NCOMMS-24-52549)

Last updated by author(s): Jul 3, 2025

Reporting Summary

Nature Portfolio wishes to improve the reproducibility of the work that we publish. This form provides structure for consistency and transparency in reporting. For further information on Nature Portfolio policies, see our Editorial Policies and the Editorial Policy Checklist.

Statistics

For all statistical analyses, confirm that the following items are present in the figure legend, table legend, main text, or Methods section.

n/a	Confirmed	
☐	☒	The exact sample size (*n*) for each experimental group/condition, given as a discrete number and unit of measurement
☐	☒	A statement on whether measurements were taken from distinct samples or whether the same sample was measured repeatedly
☐	☒	The statistical test(s) used AND whether they are one- or two-sided<br>*Only common tests should be described solely by name; describe more complex techniques in the Methods section.*
☒	☐	A description of all covariates tested
☐	☒	A description of any assumptions or corrections, such as tests of normality and adjustment for multiple comparisons
☐	☒	A full description of the statistical parameters including central tendency (e.g. means) or other basic estimates (e.g. regression coefficient) AND variation (e.g. standard deviation) or associated estimates of uncertainty (e.g. confidence intervals)
☐	☒	For null hypothesis testing, the test statistic (e.g. *F*, *t*, *r*) with confidence intervals, effect sizes, degrees of freedom and *P* value noted<br>*Give P values as exact values whenever suitable.*
☒	☐	For Bayesian analysis, information on the choice of priors and Markov chain Monte Carlo settings
☒	☐	For hierarchical and complex designs, identification of the appropriate level for tests and full reporting of outcomes
☐	☒	Estimates of effect sizes (e.g. Cohen's *d*, Pearson's *r*), indicating how they were calculated

Our web collection on statistics for biologists contains articles on many of the points above.

Software and code

Policy information about availability of computer code

Data collection	No software was used
Data analysis	CeleScope pipeline (version 1.9.0);<br>Cutadapt (version 1.17);<br>STAR software (version 2.6.1a);<br>FeatureCounts software (version 2.0.14);<br>R (version 4.1.3);<br>Seurat R package (version 2.3.4);<br>Monocle2 (version 2.8.0);<br>CellPhoneDB (version 2.0.6);<br>clusterProfiler (version 4.1)<br>GraphPad Prism (version 9.0.5)<br>FlowJo software (version 10.8.1)

For manuscripts utilizing custom algorithms or software that are central to the research but not yet described in published literature, software must be made available to editors and reviewers. We strongly encourage code deposition in a community repository (e.g. GitHub). See the Nature Portfolio guidelines for submitting code & software for further information.

Data

Policy information about availability of data

All manuscripts must include a data availability statement. This statement should provide the following information, where applicable:

- Accession codes, unique identifiers, or web links for publicly available datasets
- A description of any restrictions on data availability
- For clinical datasets or third party data, please ensure that the statement adheres to our policy

The raw sequencing data have been deposited in the GSA-Human database: https://ngdc.cncb.ac.cn/gsa-human/s/X9m197mT.

Research involving human participants, their data, or biological material

Policy information about studies with human participants or human data. See also policy information about sex, gender (identity/presentation), and sexual orientation and race, ethnicity and racism.

Reporting on sex and gender	Patient sex information was not considered in study design.
Reporting on race, ethnicity, or other socially relevant groupings	Race, ethnicity or other socially relevant groupings was not considered in study design.
Population characteristics	Information of patient characteristics was given in supplementary Table 2.
Recruitment	This study recruited pediatric patients who had been diagnosed with hemophagocytic syndrome or infectious mononucleosis, as well as healthy children who underwent routine physical examinations during the same period.
Ethics oversight	This study was approved by the Institutional Review Boards of the third Xiangya Hospital of Central South University.

Note that full information on the approval of the study protocol must also be provided in the manuscript.

Field-specific reporting

Please select the one below that is the best fit for your research. If you are not sure, read the appropriate sections before making your selection.

☒ Life sciences ☐ Behavioural & social sciences ☐ Ecological, evolutionary & environmental sciences

For a reference copy of the document with all sections, see nature.com/documents/nr-reporting-summary-flat.pdf

Life sciences study design

All studies must disclose on these points even when the disclosure is negative.

Sample size	No sample-size calculation was performed. This study performed scRNA-seq analysis on the PBMCs of 29 children, including 3 healthy volunteers (HV group), 9 patients with infectious mononucleosis (IM group) and 17 patients with EBV-HLH (HLH group). The validation group collected 7 patients newly diagnosed EBV-HLH (HLH group), 4 remission EBV-HLH patients after treatment (HLH-T group), 4 patients with infectious mononucleosis (IM group), and 6 healthy volunteers (HV group).
Data exclusions	For the 4 samples of HLH-T group, 2 cases completed both flow cytometry and tryptophan metabolomics analysis. One case only completed flow cytometry testing due to insufficient serum collected for metabolomics analysis, and one case only completed tryptophan metabolomics testing because the sample suffered from severe hemolysis, rendering flow cytometry impossible.
Replication	Results of sc-RNA-seq were validated using flow cytometry. Experiments were performed at least three times.
Randomization	No randomization was performed, as all samples were grouped according to clinically diagnosed diseases.
Blinding	Blinding was not relevant to this study, as it involved a comparative analysis of gene expression profiles in patients with different diseases.

Reporting for specific materials, systems and methods

We require information from authors about some types of materials, experimental systems and methods used in many studies. Here, indicate whether each material, system or method listed is relevant to your study. If you are not sure if a list item applies to your research, read the appropriate section before selecting a response.

Materials & experimental systems

n/a	Involved in the study	
☐	☒	Antibodies
☐	☒	Eukaryotic cell lines
☒	☐	Palaeontology and archaeology
☒	☐	Animals and other organisms
☒	☐	Clinical data
☒	☐	Dual use research of concern
☒	☐	Plants

Methods

n/a	Involved in the study	
☒	☐	ChIP-seq
☐	☒	Flow cytometry
☒	☐	MRI-based neuroimaging

Antibodies

Antibodies used

APC/Cyanine7 anti-human CD14 Antibody (BioLegend, Cat#325619);
BV421 Mouse Anti-Human CD16 Antibody (BD Biosciences, Cat#562874);
Alexa Fluor 647 Mouse Anti-Human IDO1 Antibody (D Biosciences, Cat#566648)
BV510 Mouse Anti-Human CD45(HI30) (BD Biosciences, Cat#563204);
PERCP Anti-Human HLA-DR(L243) (BioLegend, Cat#307628);
PE Mouse Anti-Human CD163(GHI/61) (BD Biosciences, Cat#560933);
BB515 Rat Anti-Human CX3CR1(2A9-1) (BD Biosciences, Cat#565902);
APC anti-human CD3 (BioLegend, Cat#317318);
FITC Mouse Anti-Human CD4(RPA-T4) (BD Biosciences, Cat#555346);
PerCP/Cyanine5.5 anti-human CD8 (BioLegend, Cat#344710);
PerCP/Cyanine5.5 anti-human CD3 Antibody (BioLegend, Cat#317336);
APC anti-human CD19 (BioLegend, Cat#302212);
RB780 Mouse Anti-HumanCD56(NCAM-1) (BD Biosciences, Cat#568763);
PE Mouse anti-NF-κB p65 (pS529) (BD Biosciences, Cat#558423);
AF647 Mouse IgG1k IsotypeControl (BD Biosciences, Cat#557732)

Validation

All antibodies used in this study are commercially available and validated by the suppliers and/or widely used in other publications.

Eukaryotic cell lines

Policy information about cell lines and Sex and Gender in Research

Cell line source(s)

THP1, U937, and NK-92 cells were from Cell Bank/Stem Cell Bank, Chinese Academy of Sciences.

Authentication

None after purchase from Cell Bank/Stem Cell Bank, Chinese Academy of Sciences.

Mycoplasma contamination

Cell lines were not tested for mycoplasma.

Commonly misidentified lines
(See ICLAC register)

Not applicable

Plants

Seed stocks

Plants were not used in this study.

Novel plant genotypes

Plants were not used in this study.

Authentication

Plants were not used in this study.

Flow Cytometry

Plots

Confirm that:

- [x] The axis labels state the marker and fluorochrome used (e.g. CD4-FITC).
- [x] The axis scales are clearly visible. Include numbers along axes only for bottom left plot of group (a 'group' is an analysis of identical markers).
- [x] All plots are contour plots with outliers or pseudocolor plots.
- [x] A numerical value for number of cells or percentage (with statistics) is provided.

Methodology

Sample preparation	PBMCs were stained with fluorochrome-labeled antibodies for the following surface markers: CD45, CD3, CD4, CD8, CD56, CD19, HLA-DR, CD163, CX3CR1, CD14 and CD16. For intracellular staining, cells were fixed and permeabilized according to the manufacturer's instructions (Cytofix/Cytoperm and Perm/Wash Buffer, BD Biosciences) and stained for 50 min on ice for IDO1 and p-p65.
Instrument	BD FACSCelesta
Software	FlowJo software (version 10.8.1)
Cell population abundance	NO flow cytometry sorting.
Gating strategy	cMo cells were gated from CD14+CD16- monocytes; iMo cells were gated from CD14+CD16+ monocytes; ncMo were gated from CD14dimCD16+ monocytes.

- [x] Tick this box to confirm that a figure exemplifying the gating strategy is provided in the Supplementary Information.
